# Electrocatalytic Conversion of Renewable Electricity—What Molecules are More Promising as Energy Storage Media?

**DOI:** 10.1002/tcr.202500012

**Published:** 2025-09-09

**Authors:** Jacob Johny, Sayed M. El‐refaei, Justus Masa, Aleksandar R. Zeradjanin

**Affiliations:** ^1^ Electrochemistry Group Max Planck Institute for Chemical Energy Conversion Stiftstrasse 34–36 45470 Mülheim an der Ruhr Germany

**Keywords:** carbon cycle, electrocatalysis, energy efficiency, hydrogen cycle, nitrogen cycle

## Abstract

An analysis is conducted with the intention to clarify which molecules are more promising as renewable electricity storage media, taking into consideration some basic parameters like theoretical and practical voltage, theoretical energy density, etc. The central aspect of analysis is to apply sufficiently simple, but relevant criterion, the minimum cost of electricity required to produce a specific quantity of chemical energy storage medium, in relation to the prevailing market prices of the produced chemicals. Therefore, the study analyzes the cost of electrical energy needed to selectively convert CO_2_ into specific molecules such as, CO, CH_3_OH, and CH_4_, among others, water into hydrogen, and nitrogen into ammonia, by considering both idealized and more realistic operational conditions. The results show that in the case of energy carriers that are too expensive to be generated under idealized conditions, further detailed analysis of other production factors is inconsequential. The production of hydrogen, formic acid, and syngas (CO and H_2_) as energy carriers is economically feasible under realistic operational conditions. It is also conceivable that further electricity‐to‐chemical conversion efficiency gains can be realized for these molecules thus underscoring the need for their prioritization.

## Graphical abstract

The minimum cost of electricity consumed for energy medium generation, under realistic and idealized conditions, is instrumentalized as discriminatory criterion, in relation to the prevailing market prices of the produced chemicals. Fundamental reason on why selectivity control becomes challenging is proposed when we move from small toward more complex energy storage molecules.

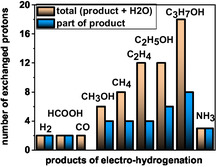

## 1 Introduction

One of the greatest technological challenges in this era is how to supply a sufficient amount of electricity in order to respond to increasing demands for electricity in industry, transportation, households, and the growing infrastructure for utilization of artificial intelligence (AI), among others. Global electricity consumption today is around 30 000 TWh,^[^ ^1^ ^]^ which is above 19% of overall energy demand.^[^ ^2^, ^3^ ^]^ Both global overall energy demand as well as global electricity consumption are increasing; however, the latter increases at a higher rate than the former.^[^ ^4^, ^5^ ^]^ The encouraging fact is that the amounts of energy originating from renewable sources (e.g., solar, wind, geothermal, etc.) are orders of magnitude higher than our actual needs.^[^ ^6^ ^]^ Therefore, devices/systems that can transform energy from renewable sources into electricity (e.g., photovoltaics (PVs) or wind turbines) and then transmit it through the grid directly toward consumers are of strategic importance for the future. Progress in this direction is evident globally.^[^ ^7^ ^]^ At the same time, renewable energy sources fluctuate during 24 h in a way that is out of synchrony with energy demand, so the major challenge is to design processes and devices that will allow effective storage of electricity and usage of stored energy according to demand, again in the form of electricity.^[^ ^8^ ^]^ For the electricity storage processes, essential are electrode reactions at which interfacial charge transfer (i.e., usually at solid/liquid interfaces) is integral part of ongoing redox chemical reaction, at room temperature, ambient pressure, and under flexible control of reaction rate by electrode potential. The ideal case would be if storage and usage apply to single devices, as in the case of rechargeable batteries. However, batteries are closed systems (i.e., exchange energy, but not matter with its surroundings) with relatively modest energy density (i.e., the amount of energy stored per unit mass or volume), where ions participate in bulk processes (e.g., intercalation). Therefore, more relevant are systems with higher energy densities, where, also, due to mobile liquid phase, rearranging of chemical bonds and generation of small molecules (e.g., H_2_, CH_4_, NH_3_) at electrified solid/liquid interface proceeds in a more flexible manner, predominantly at the electrode surface, meaning without significant distortion of the electrode structure.

Despite the advantages of the mentioned chemical energy conversion and storage processes at electrified interfaces over classical thermally driven heterogeneous or homogeneous reactions, they require stable electrocatalysts that will dictate energy efficiency and selectivity of the conversion process.^[^ ^9^ ^]^ Practically, all the reactions used as basis of renewable electricity storage are complex with mechanisms that are still under investigation.^[^ ^10^ ^]^ In this work, we analyzed the chemical energy conversion systems at electrified interfaces (i.e., electrochemical conversion systems) that would be more realistic from the point of process efficiency. In previous research, substantial efforts were made to give detailed and relevant technoeconomic analysis of renewable hydrogen production,^[^ ^11^ ^]^ electrochemical CO_2_ reduction,^[^ ^12^ ^]^ and green ammonia production.^[^ ^13^ ^]^ The reports contained detailed information and complex analysis that is relevant, nevertheless, it can be observed that the lack of simplicity compromised clarity of the main conclusions.^[^ ^11^ ^]^ Also, different studies suggest different products to be economically feasible, due to different assumptions and different elements of the used technoeconomic models.^[^ ^12^, ^14^ ^]^ Predictions spread from very optimistic, considering the majority of products,^[^ ^14^ ^]^ toward those who predict that only a few products are economically feasible,^[^ ^12^, ^15^ ^]^ up to predictions that all products have very low feasibility.^[^ ^16^ ^]^ Different viewpoints could also be partially explained by different experimental results or different literature datasets used to feed the parameters of the technoeconomic models. Therefore, we will focus on a minimum of possible parameters, but still sufficient to give reliable, insightful answers to the question of what molecules are more promising as energy storage media. A key distinctive outcome of this work is that, even if we cannot decisively determine what energy media are most viable, we can definitely obtain a reliable picture on evidently nonfeasible energy media.

## 2 Results and Discussion

### 2.1 Water Electrolysis

A very versatile technology to store renewable electricity is water electrolysis, where the hydrogen evolution reaction (HER) proceeds at the cathode and the oxygen evolution reaction (OER) proceeds at the anode.^[^ ^10^, ^17^, ^18^ ^]^ It is a mature technology, considered to be the cornerstone of the hydrogen economy, which, however, is still not efficient enough to be recognized as a state‐of‐the‐art technological solution.^[^ ^19^, ^20^ ^]^ In the past, alkaline water electrolysis was favored due to the possibility of using cheap electrodes and electrolyte materials.^[^ ^17^ ^]^ Nowadays, acidic polymer electrolyte membrane (PEM) electrolyzers are more attractive as they allow much higher current densities despite the necessity to use expensive and rare noble metals as components of the electrode materials.^[^ ^17^ ^]^ The two main reactions occurring in an acidic (pH = 0) electrolyzers are

(R1) $$\;2H^{+}+2e^{-}\;⇌{\text{ H}}_{2}\;E{}^{\circ}\;=\;0.00\text{ V vs}.\text{ SHE}$$

(R2) $$2H_{2}\text{O }⇌O_{2}+4H^{+}+4e^{-}\;E{}^{\circ}\;=\;1.23\text{ V vs}.\text{ SHE}$$

Similar equations can be written for alkaline (pH = 14) electrolyzers

(R3) $$2H_{2}O+2e^{-}\;⇌{\text{ H}}_{2}+2{\text{OH}}^{-}\;E{}^{\circ}\;=\;-0.83\text{ V vs}.\text{ SHE}$$

(R4) $$4{\text{OH}}^{-}\;⇌{\text{ O}}_{2}+\;2H_{2}O+2e^{-}\;E{}^{\circ}\;=\;0.40\text{ V vs}.\text{ SHE}$$

The potentials are expressed on the standard hydrogen electrode (SHE) scale to clearly indicate the impact of pH value on redox potentials at the absolute potential scale.^[^ ^21^ ^]^ In the case of pH‐dependent reactions like HER and OER, for every unit change in pH, the equilibrium redox potential is shifted by 59 mV. The same reaction has always more positive potential in acidic media than in alkaline media. In both media, the thermodynamic driving force necessary to conduct the process of water electrolysis is voltage of minimum 1.23 V at standard conditions (temperature of 25 °C and pressure of 1 bar). In reality, voltage is significantly larger due to various losses (ohmic drops, mass transport limitations, kinetic limitations of electrode reactions, etc.). The voltage that will be established at an electrochemical reactor for water electrolysis, at defined current density, will essentially determine the energy efficiency of H_2_ production.

Projections of officials from European Commission are that the price of hydrogen till year 2030 will be below 2 Euro kg^−1^ or till year 2050 will be below 1 Euro kg^−1^.^[^ ^22^ ^]^ The total costs of hydrogen production, excluding storage and transport, depend on multiple parameters, including: capital investment, operational expenses, equipment efficiency, scale of production, etc. Translating that to scientific and engineering challenges we can say that the most important tasks are: 1) to reduce the amount of noble metals used as electrocatalysts (or ideally to eliminate them completely),^[^ ^23^ ^]^ 2) to operate under high current densities (i.e., higher turnover of product per unit area of electrode surface),^[^ ^24^, ^25^ ^]^ 3) to reduce energy/electricity consumption necessary to run electrode reactions,^[^ ^26^ ^]^ and 4) to ensure that materials used as electrocatalyst, support materials, etc. can endure at operational conditions on the timescale of several years without significant loss in performance, despite the intermittent nature of renewable electricity sources.^[^ ^27^ ^]^ If we completely eliminate noble metals from the system (i.e., using alkaline electrolyzers) and significantly enlarge operating current densities, still there is a minimal amount of energy necessary to produce defined amount of H_2_ as a unavoidable consequence of thermodynamics of electrode reactions, stoichiometry of electrode reactions and Faraday's law (i.e., quantifies proportionality between mass and charge of species participating in electrode reactions). Minimum energy required to split water by electric current is given by Equation (1):

(1) ΔG°=−nFU°

where Δ*G°* is the change of molar free energy during molecular hydrogen generation dissolved in electrolyte (Equation R1 or R3) and molecular oxygen generation dissolved in electrolyte (Equation R2 or R4), generated from liquid water (i.e., observe sum of Equation R1 and R2 or sum of Equation R3 and R4); *n* is the number of exchanged electrons to generate one molecule of H_2_; and *F* is the Faraday constant; *U°* is the theoretical voltage or equilibrium electrode potential of cathode (Equation R1 or R3) minus equilibrium electrode potential of anode (Equation R2 or R4). Water splitting is an endergonic or nonspontaneous (ΔG° > 0) process (Equation 2):

(2) $$\Delta G{}^{\circ}\;=\;-\;2\;\times \;96500{\text{ Cmol}}^{-1}\;\times \;\left(-1.23\text{ V}\right)=237.\;4{\text{ kJ mol}}^{-1}$$

In other words, for water electrolysis, we need to invest a minimum of 237.4 kJ mol^−1^ to drive the process. This thermodynamically defined value at equilibrium conditions (i.e., equilibrium voltage) is the actual amount of electrical energy stored by rearranging chemical bonds of 1 mol of reactant (water) into 1 mol of product (hydrogen). Additional electrical energy consumed for conversion of 1 mol of water into 1 mol of hydrogen, due to intrinsic kinetic reasons, mass transport limitations, ohmic drops, etc., will be exchanged with the environment in the form of Joule's heat. If we want to calculate how much energy is required for 1 kg of H_2_, we just divide free energy of water splitting with molar mass of H_2_, obtaining 118.7 kJ g^−1^. In some literature sources, the value of 143 kJ g^−1^ is often reported,^[^ ^28^ ^]^ that, instead of being based on free energy of water electrolysis 237.4 kJ mol^−1^ and the equilibrium voltage of 1.23 V, is erroneously based on the value of the enthalpy for water electrolysis of 285.8 kJ mol^−1^ and thermoneutral voltage of 1.48 V (i.e., voltage at which net exchange heat with environment is zero). However, only the amount of electricity stored at the equilibrium potential that corresponds to the free energy of water electrolysis can be recovered back as electricity. Considering that 1 kWh is equivalent to 3.6 MJ, a minimum of 33 kWh of electricity is required to produce 1 kg of H_2_. This value is universal and is basis on which any other costs can be added. Therefore, before any technoeconomic model is considered, it is necessary to compare the cost of the minimum amount of electricity required to produce 1 kg of H_2_ with the average market price of 1 kg of H_2_. The market price of hydrogen obtained in the conventional way from the steam reforming of methane is 1.0–2.0 Euro kg^−1^, while 2.0 Euro kg^−1^ will be taken as the reference value for further analysis.^[^ ^29^ ^]^

It is important to keep in mind that the minimum energy required to obtain 1 kg of H_2_ depends on the theoretical voltage and the charge required to produce 1 kg of H_2_. The charge that is required to produce 1 kg of H_2_ can be obtained from Faraday's law (Equation 3):

(3) $$Q=\frac{mnF}{M}$$

where, *Q—*charge used to drive electrolysis, that is, defined by electric current flowing through the interface of ionic and electronic conductor in finite time; *m—*mass of product (i.e., H_2_) and *M—*molar mass of H_2_. Therefore, the charge necessary to produce 1 kg of H_2_ by electrolysis is given by Equation (4), for the assumption that there are no side reactions:

(4) $$Q=1\text{ kg }\times \;\frac{2\;\times \;96\;500{\text{ Cmol}}^{-1}}{2\;\times 10^{-3}{\text{ kgmol}}^{-1}}=\;96.5\;\times \;10^{6}\text{ C }$$

**Figure 1:**
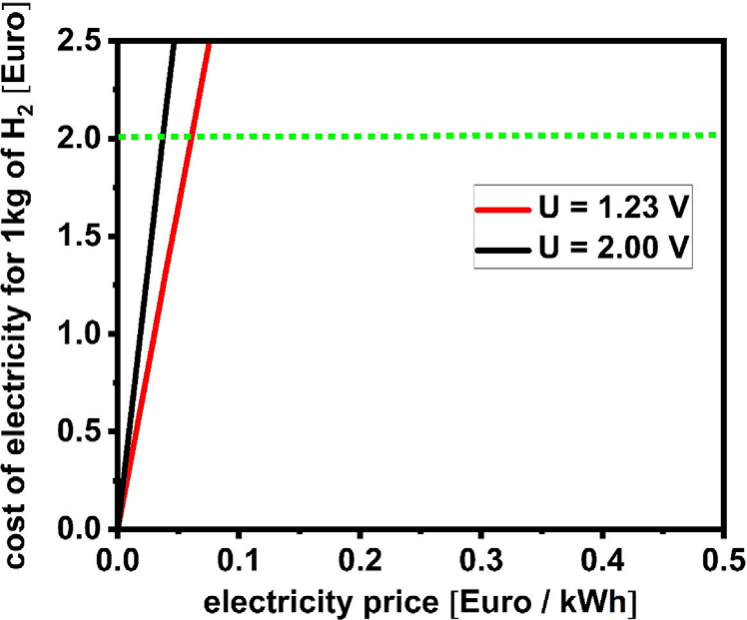
Cost of electricity used to produce 1 kg of H_2_ as a function of the electricity price of 1 kWh of renewable electricity at the theoretical voltage for water splitting (red line) and at realistic voltage for water splitting (black line). The dotted green line indicates the targeted price of hydrogen. Space between the red and black solid lines, below the green dotted line indicates where the kinetics of water splitting can play a role in enhancing energy efficiency and reducing the cost of electrolysis.

Evidently, the charge necessary to produce 1 kg of H_2_ by electrolysis is strongly dependent on the ratio between the molar mass of H_2_ and the number of exchanged electrons in the electrode reaction or we can say that the minimum energy to produce 1 kg of H_2_ is essentially impacted by theoretical voltage and by the ratio between molar mass of H_2_ and number of exchanged electrons. This is very important to have in mind because it indicates how, besides theoretical voltage, the stoichiometry of electrode reactions strongly impacts the energetics of water splitting or any other energy conversion processes. **Figure** **1** shows the dependence of the cost of electricity required to produce 1 kg of H_2_ on the price of renewable electricity and voltage for water electrolysis. Price range for electricity was chosen to cover the range of renewable electricity prices^[^ ^30^ ^]^ as well to consider major part of the price range of industrial electricity worldwide.^[^ ^31^ ^]^ If the price of 1 kWh of electricity is above 6 Eurocent, the cost of electricity required to produce 1 kg of H_2_ would be above 2 Euro even if water electrolysis was conducted at equilibrium voltage. Under the working conditions (i.e., transient current load related to source of renewable electricity), the energetics of water splitting are impacted by the two aforementioned fixed parameters (i.e., theoretical voltage and ratio between molar mass of H_2_ and number of exchanged electrons) and two variable parameters including additional voltage due to various energy losses (i.e., kinetic overpotentials, diffusion overpotentials, ohmic drops, etc.) and cost of used electricity. Nowadays, the voltage for water electrolysis at working conditions is around 2 V.^[^ ^17^ ^]^ To take into consideration hydrogen production under operational voltage of around 2.0 V as it is today, the price of 1 kWh of electricity has to be below 3 Eurocent. Theoretically, voltage can be minimized down to the thermodynamic value of 1.23 V. So, the cost of electricity used to produce 1 kg of H_2_ can be reduced by accelerating reaction macrokinetics in the ideal case by ≈40%. That is significant, although almost impossible to be achieved. Besides reducing voltage further, what is of major relevance is that the key challenge for improvement of the energy efficiency of hydrogen production lies in reducing the price of renewable electricity. By analyzing the prices of electricity for enterprises worldwide, we can see that hydrogen production would be economically feasible in only a few countries in the world, and notably, these are countries that currently have a low intensity of electricity production.^[^ ^31^ ^]^ In the industrially developed countries, which are large electricity producers and consumers, it is possible that the price of 1 kWh of electricity is between 3.5 and 5 Eurocent, but these prices are heavily subsidized by the state, typical of gigantic electricity consumers that require beyond 150 GWh per year, like aluminum smelters, copper smelters, and chlor‐alkali technology.^[^ ^32^ ^]^ The electricity prices for enterprises in China, USA, and India, as the three countries that produce more than half of electricity worldwide, are still way too high to respond to mentioned requirements.^[^ ^31^ ^]^ The encouraging fact is that electricity generated from renewable energy sources which covers ≈30% of electricity generated worldwide,^[^ ^33^ ^]^ has the potential to be significantly cheaper than electricity obtained from other sources (i.e., fossil fuels, nuclear fuels, or geothermal sources).^[^ ^30^ ^]^ This is especially true for solar and wind, which are responsible for ≈13% of electricity generated worldwide.^[^ ^33^ ^]^ Besides the significant drop in the cost of electricity obtained from concentrated solar and offshore wind, even more significant is the drop of renewable electricity cost obtained from onshore wind and PV.^[^ ^30^ ^]^ If we observe the developments over a long term related to existing installed power, it seems that PV shows a tendency to be the leading technology for generation of renewable electricity.^[^ ^34^ ^]^ On the one hand, this is essentially due to the infinite potential of the sun as an energy source, and on the other hand, due to the fact that the anticipated efficiency limitations of PV (i.e., Shockley–Queisser limit) can be exceeded with multijunction cells^[^ ^35^, ^36^ ^]^ while those associated with wind turbines (i.e., Betz's law) seems to be more rigid.^[^ ^37^ ^]^ Therefore, the exact surface area of the Earth that should be covered with PV is still an open question. Despite discouraging moments where the average price of hydrogen fuel at publicly accessible filling stations in the US in 2024 reached over 30 Euro per kilogram, we have promising examples in South Korea and Japan, where the price was around 7 Euro per kilogram.^[^ ^38^ ^]^ It is also interesting to observe the existing disparity in the evolution of electricity prices during the last seven years in different regions of the world.^[^ ^39^ ^]^ While electricity prices in the US experienced modest increase, the increase was relatively sharp in the EU.^[^ ^39^ ^]^ At the same time, in India, there is no significant change observable, while in China, there was a noticeable drop in electricity price.^[^ ^39^ ^]^ In summary, in relation to hydrogen, we can say that it will require some time until it becomes feasible. Reducing electrolysis voltage (e.g., reaching a voltage of 1.5 V) would be of significance, but it is not the main challenge we are facing. The most important would be that price of electricity from PVs, which is currently about 0.036 Euro per kWh,^[^ ^40^ ^]^ goes below 0.02 Euro per kWh.

### 2.2 Electrolytic CO_2_ Conversion

In comparison to water electrolysis, which is a mature technology, electrolytic CO_2_ conversion is an emerging technology under development. Very intensive activities have been undertaken during the last 10–15 years with a focus on various possible products of CO_2_ electrolysis, known also as electrochemical CO_2_ reduction.^[^ ^41^ ^]^ When discussing electrochemical CO_2_ reduction, we consider usually the following reactions and concomitant products:

(R5) $${\text{CO}}_{2}\;+\;2H^{+}+2e^{-}\;⇌\text{ HCOOH }E{}^{\circ}\;=\;-\;0.20\text{ V vs}.\text{ SHE}$$

(R6) $${\text{CO}}_{2}\;+\;2H^{+}+2e^{-}\;⇌\text{ CO}+{\text{ H}}_{2}\text{O }E{}^{\circ}\;=\;-\;0.11\text{ V vs}.\text{ SHE }$$

(R7) $${\text{CO}}_{2}\;+\;6H^{+}+6e^{-}\;⇌{\text{ CH}}_{3}\text{OH}+{\text{ H}}_{2}\text{O }E{}^{\circ}\;=\;0.030\text{ V vs}.\text{ SHE}$$

(R8) $${\text{CO}}_{2}\;+\;8H^{+}+8e^{-}\;⇌{\text{ CH}}_{4}+\;2H_{2}\text{O }E{}^{\circ}\;=\;0.17\text{ V vs}.\text{ SHE}$$

(R9) $$2{\text{CO}}_{2}\;+\;12H^{+}+12e^{-}\;⇌{\text{ C}}_{2}H_{4}+\;4H_{2}\text{O }E{}^{\circ}\;=\;0.064\text{ V vs}.\text{ SHE }$$

(R10) $$2{\text{CO}}_{2}\;+\;12H^{+}+12e^{-}\;⇌{\text{ C}}_{2}H_{5}\text{OH}+\;3H_{2}\text{O }E{}^{\circ}\;=\;0.084\text{ V vs}.\text{ SHE }$$

(R11) $${\text{3CO}}_{2}\;+\;18H^{+}+18e^{-}\;⇌{\text{ C}}_{3}H_{7}\text{OH}+\;5H_{2}\text{O }E{}^{\circ}\;=\;0.095\text{ V vs}.\text{ SHE }$$

**Figure 2:**
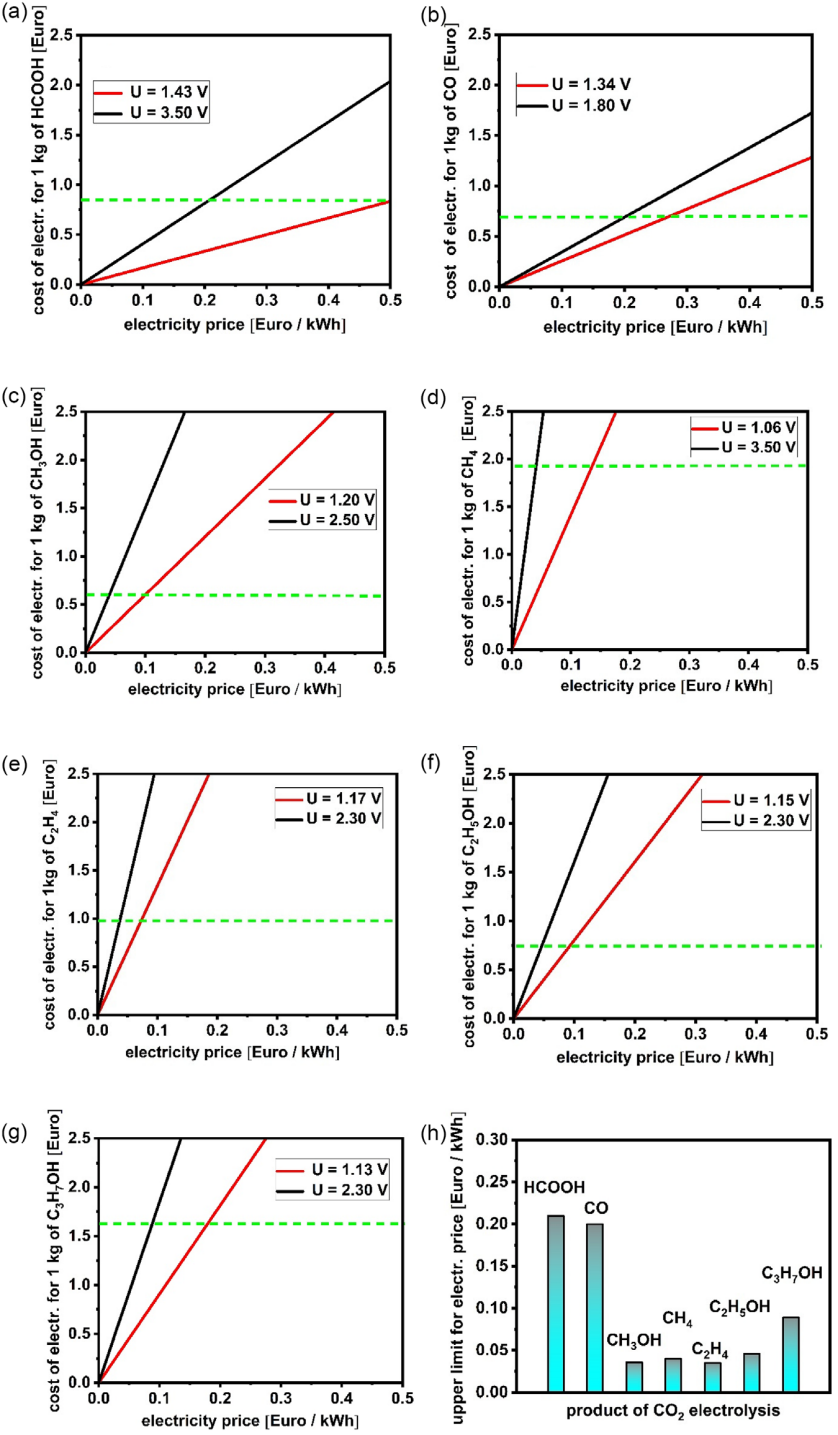
Cost of electricity used to produce 1 kg of H_2_ as a function of the price of 1 kWh of renewable electricity given at the theoretical voltage for electrochemical CO_2_ reduction (red line) and at a realistic voltage for electrochemical CO_2_ reduction (black line). Green dotted line indicates the market price of the products that should be generated by electrochemical CO_2_ reduction including: a) HCOOH, b) CO, c) CH_3_OH, d) CH_4_, e) C_2_H_4_, f) C_2_H_5_OH, and g) C_3_H_7_OH, h) upper limit for the price of 1 kWh of renewable electricity if electrolysis is conducted at a realistic voltage for each product.

Typical products of electrochemical CO_2_ reduction in older literature are C_1_, C_2_, and C_3_ compounds.^[^ ^42^ ^]^ In more recent literature, C_4+_ products have also been reported,^[^ ^43^, ^44^ ^]^ although as a matter of fact, selectivity control appears to be experimentally a very challenging task, even in the case of simple C_1_ products,^[^ ^42^ ^]^ especially if they require the exchange of more than two electrons in the redox process. Having in mind that the reaction at the counter electrode is always OER (Equation R2), the thermodynamic voltage necessary to generate each of the products of CO_2_ reduction in reactions 5–11 can be derived. Evidently, the theoretical voltage for CO_2_ reduction will be in the range 1.03–1.40 V, which is roughly ±200 mV in comparison to the theoretical voltage of water splitting. All the reactions of CO_2_ reduction (i.e., Equation (R5), (R6), (R7), (R8), (R9), (R10), (R11)) as well as HER (Equation R1) are thermodynamically possible. In experimental works, CO_2_ reduction in acidic media is usually avoided to minimize the impact of HER; meanwhile, CO_2_ is transformed into carbonate salts in alkaline media. Therefore, electrochemical CO_2_ reduction is typically carried out in neutral media despite well‐known challenges, including: low concentration of dissolved reactant, gas‐bubble evolution, changes of cathode surface composition and structure due to deposition of impurities dissolved at the anode during OER (i.e., independently from the nature and properties of the separator), local interfacial pH, etc. Taking into consideration the fact that a faradaic efficiency (FE) of less than 100% additionally increases the production cost per kilogram of product due to increase in energy consumption as well as due to additional costs related to product separation, we will intentionally consider an ideal case where we obtain every product described in the reactions Equation ((R5), (R6), (R7), (R8), (R9), (R10))–(R11) with 100% selectivity and excluding any other costs of electrochemical CO_2_ conversion (e.g., CO_2_ capture, product separation, etc.) as a point of reference (**Figure** **2**). Comparison of electrolysis costs will be made between equilibrium voltage and realistic operational voltage and in relation to the market price of the respective products.

**Formic acid**—considering the commercial price of 1 kg of HCOOH, which is around 0.84 Euro, if electrolysis would be conducted at a realistic voltage of 3.5 V,^[^ ^45^ ^]^ the acceptable price of electricity should be below 0.21 Euro per kWh (Figure 2a). It is common that CO_2_ electroreduction to HCOOH has selectivity over 80% at a current density of at least 200 mA cm^−2^.^[^ ^46^ ^]^ With the current price of renewable electricity from PV of around 0.036 Euro kWh^−1^, we could say that HCOOH is a very promising solution. Values of realistic operational voltage,^[^ ^45^ ^]^ selectivity (or faradaic efficiency),^[^ ^46^ ^]^ and current density^[^ ^46^ ^]^ are given for every energy storage medium in **Table** **1**, while commercial prices of energy medium^[^ ^45^ ^]^ and acceptable price of electricity are given in **Table** **2**.

**Table 1:** Parameters determining efficiency of energy conversion reactions at electrified interfaces including: number of exchanged electrons (*n*_e_), molar mass (*M*), charge that has to pass through electrode/electrolyte interface to form 1 kg of product (*Q*), theoretical voltage (*U*_theo_), practical voltage at operational current density (*U*_prac_), voltage efficiency (i.e., equivalent to energy efficiency) is defined as the ratio between theoretical voltage and the practical electrolysis voltage (*ε*_*U*_), faradaic efficiency or selectivity (FE), current density (*j*). Values for (*U*_prac_), (FE) and (*j*) taken from references indicated in the main text designated to each energy storage medium.

	*n*_e‐_	*M* [gmol^−1^]	(*M*/*n*_e‐_) [gmol^−1^e^−1^]	*Q* [MCkg^−1^]	*U*_theo_ [V]	*U*_prac_ [V]	*ε*_*U*_	FE	*J* [Acm^−2^]
H_2_	2	2	1	96.5	1.23	2.00	0.62	1.00	2.00
HCOOH	2	46	23	4.2	1.43	3.50	0.41	0.90	0.20
CO	2	28	14	6.9	1.34	1.80	0.74	0.95	0.60
CH_3_OH	6	32	5.3	18.2	1.20	2.50	0.48	0.35	0.30
CH_4_	8	16	2	48.2	1.06	3.50	0.30	0.80	0.10
C_2_H_4_	12	28	2.3	42.0	1.17	2.30	0.51	0.60	0.60
C_2_H_5_OH	12	46	3.8	25.4	1.15	2.30	0.50	<0.30	0.25
C_3_H_7_OH	18	60	3.3	29.2	1.13	2.30	0.49	0.15	<0.050
NH_3_	3	17	5.7	16.9	1.17	2.00	0.58	0.15 ?	<0.050

**Table 2:** Parameters determining the amount of energy stored per unit mass of the respective medium as well as parameters determining the unit cost of the stored energy, via energy conversion reactions at electrified interfaces including: theoretical specific energy or energy stored in 1 kg of medium at the theoretical voltage (*W*_theo_), practical specific energy or energy consumed for storage of 1 kg of the medium at the practical voltage (*W*_prac_), theoretical volumetric energy or energy stored in 1 L of medium at the theoretical voltage (*W*_theo,vol_), number of protons that actually participate in building of molecule of product (*n*_*H*_), market price of 1 kg of energy storage medium (m. price),^[^ ^45^ ^]^ upper acceptable electricity price given in the range of values from theoretical voltage up to operational voltage (price el.).

	*W*_theo_ [kWh kg^−1^]	*W*_prac_ [kWh kg^−1^]	*W*_theo,vol_ [kWh L^−1^]	*n*_*H*_	m. price [Euro kg^−1^]	Price el. [Cent kWh^−1^]
H_2_	33.0	53.7	0.0028	2	2.00	6.1–3.7
HCOOH	1.7	4.1	2.0	2	0.84	49.4–20.5
CO	2.6	3.4	0.0030	0	0.68	26.2–11.7
CH_3_OH	6.1	12.6	4.8	4	0.59	9.7–4.7
CH_4_	14.2	46.9	0.0093	4	1.92	13.5–4.1
C_2_H_4_	13.6	26.8	0.018	4	0.97	7.1–3.6
C_2_H_5_OH	8.1	16.2	6.4	6	0.73	9.0–4.5
C_3_H_7_OH	9.3	18.7	7.4	8	1.62	17.4–8.7
NH_3_	5.5	9.4	0.0039	3	0.46	8.4–4.9

**Carbon monoxide**—very interesting for analysis is CO, as a building block in the chemical industry. Considering the current commercial price of around 0.68 Euro for 1 kg of CO,^[^ ^45^ ^]^ if electrolysis would be conducted at a realistic voltage of 1.8 V, the acceptable price of electricity should be below 0.20 Euro per kWh (Figure 2b). While the voltage for CO production could deviate significantly in the literature, the selectivity was improved during the years and is now reported in some studies to be over 90%, reaching 100% at a current density of 400 mA cm^−2^.^[^ ^47^ ^]^ This makes CO to be also a very promising solution if we consider the current prices of renewable electricity. Note that both HCOOH and CO exchange only two electrons and two protons, suggesting only one intermediate, which makes selectivity control of the reaction to be relatively easy.

**Methanol**—CO_2_ conversion to CH_3_OH is related to exchanging six electrons and six protons, suggesting probably five intermediates, which makes it a very challenging case to control the reaction selectivity. Considering commercial price of 1 kg of CH_3_OH, which is around 0.59 Euro, if electrolysis would be conducted at realistic 2.50 V,^[^ ^45^ ^]^ acceptable price of 1 kWh should be below 0.036 Euro. This makes CH_3_OH a very challenging solution, especially if we consider that at a current density of 300 mA cm^−2^, the selectivity is only around 50%^[^ ^45^ ^]^ or probably even less.^[^ ^14^ ^]^

**Methane**—considering commercial price of 1 kg of CH_4_, which is around 1.92 Euro, if electrolysis would be conducted at realistic 3.5 V,^[^ ^45^ ^]^ acceptable price of 1 kWh should be below 0.04 Euro. This seems to be a similar situation like in the case of methanol, however, to convert CO_2_ to CH_4_, despite the selectivity being higher than in the case of CH_3_OH, reaching close to 80%, the overpotential for the conversion of CO_2_ to CH_4_ is increasing drastically with the current density.^[^ ^48^ ^]^ At a current density of 100 mA cm^−2^, the reported voltage was 3.50 V, while at 250 mA cm^−2^ it increased to 4.25 V.^[^ ^48^ ^]^ Evidently, electrocatalytic conversion of CO_2_ to CH_4_ is very challenging, lacking flexibility to be operated at high current densities at a reasonable voltage. Importantly, the reaction mechanism involves exchange of eight electrons and eight protons, suggesting probably seven possible intermediates, making it even more challenging to control the reaction pathway than in the case of CH_3_OH.

**Ethylene**—considering commercial price of 1 kg of C_2_H_4_, which is around 0.97 Euro, if electrolysis would be conducted at realistic 2.3 V,^[^ ^45^ ^]^ acceptable price of kWh should be below 0.035 Euro. The selectivity is estimated to be close to 90% at a current density of 200 mA cm^−2^, while at 600 mA cm^−2^ it is significantly lower, close to 60%.^[^ ^14^ ^]^ There are studies that are less optimistic,^[^ ^49^ ^]^ for example, suggesting that at a voltage of 2.4 V and a current density of 110 mA cm^−2^, the selectivity is around 70%.^[^ ^49^, ^50^ ^]^ Having all this in mind, CO_2_ conversion to C_2_H_4_ is also recognized as a very challenging case in our study. Process is related with exchange of twelve electrons and twelve protons, where the mechanism includes C‐C coupling, which makes it even more challenging to control reaction selectivity than in the case of methane.

**Ethanol**—reaction mechanism requires the exchange of twelve electrons and twelve protons, including C‐C coupling, like in the case of ethylene, making it very challenging to control reaction selectivity. Considering commercial price of 1 kg of C_2_H_5_OH, which is around 0.73 Euro, if electrolysis would be conducted at realistic 2.3 V,^[^ ^45^ ^]^ acceptable price of kWh should be below 0.046 Euro. This seems to be a bit more promising than in the case of CH_3_OH, CH_4_, and C_2_H_4_; however, the selectivity is very low. At a current density of 250 mA cm^−2^ the selectivity is slightly below 30%.^[^ ^45^ ^]^ Therefore, CO_2_ conversion to C_2_H_5_OH when considering all this is still very unlikely to be feasible in immediate future.

**Propanol**—despite the relatively poor availability data on the voltage for CO_2_ conversion to C_3_H_7_OH the following conclusions could be made: considering the commercial price of 1 kg of C_3_H_7_OH, which is around 1.62 Euro, if electrolysis would be conducted at a realistic voltage of 2.3 V,^[^ ^45^ ^]^ the acceptable price for 1 kWh of electricity should be below 0.089 Euro, which makes it an interesting case from that perspective. The process is associated with the exchange of eighteen electrons and eighteen protons, where the mechanism includes C‐C‐C bond formation, suggesting even higher complexity than in the case of ethylene and ethanol, which makes it very challenging to control reaction selectivity. Experimental results show that it is difficult to achieve selectivity even close to 20% and that applied current densities are significantly lower than 100 mA cm^−2^,^[^ ^14^, ^51^, ^52^ ^]^ making electrocatalytic CO_2_ conversion to C_3_H_7_OH very unlikely to be a feasible solution in the immediate future.

### 2.3 Electrolytic Synthesis of NH_3_

Besides hydrogen, hydrocarbons, and alcohols, what attracted substantial attention during last decade is ammonia as energy storage medium.^[^ ^53^, ^54^, ^55^ ^]^ Direct electrolytic synthesis of ammonia from abundant reactants would assume that molecular nitrogen extracted from air would be reduced at cathode by electrons and protons released from anode, where water oxidation occurs (i.e., OER). Theoretical voltage of this process is around 1.17 V. Reaction at the cathode is shown with Equation (R12).

(R12) $$N_{2}\;+\;6H^{+}+6e^{-}\;⇌\;2{\text{ NH}}_{3}\;\;\;\;\;E{}^{\circ}\;=\;0.060\text{ V vs}.\text{ SHE}$$

**Figure 3:**
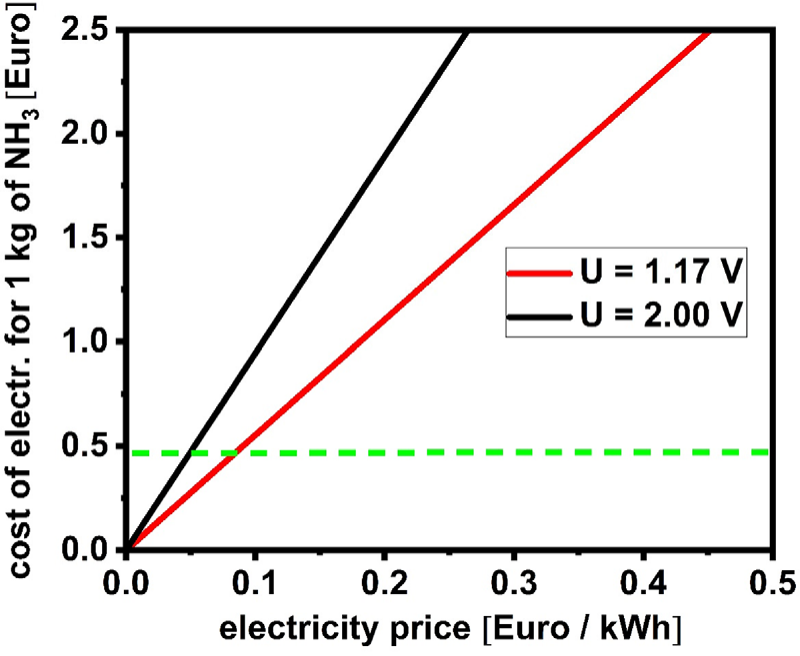
Cost of electricity used to produce 1 kg of NH_3_ as function of the price of kWh of renewable electricity given at theoretical voltage for electrolytic ammonia generation (red line) and at realistic voltage for electrolytic ammonia generation (black line). Green dotted line indicates market price of NH_3_.

For the generation of one molecule of NH_3_, three protons and three electrons, which should place this conversion process to be more complicated than the generation of HCOOH or CO, but less demanding than the generation of CH_3_OH, and therefore, potentially promising. This should be valid for both electricity consumption and for the selectivity control. Despite the existence of some comprehensive reviews on electrosynthesis of NH_3_, they generally lack systematic data on selectivity and voltage for various electrode materials, electrolytes, reactor types, etc.^[^ ^13^ ^]^ One of the reasons is that it is difficult to have an accurate and reliable experimental protocol for selectivity evaluation.^[^ ^56^ ^]^ Existing studies investigated NH_3_ electrosynthesis in the voltage range from 1.5 to 2.5 V.^[^ ^57^ ^]^ Interestingly, below a voltage of 1.55 V, it was not possible to get a stable current response.^[^ ^58^ ^]^ At the same time, NH_3_ generated at a voltage of 1.8 V and selectivity of 100% corresponds to 460 kJ mol^−1^ or 8.5 kWh kg^−1^, which is comparable to the energy consumed in the state‐of‐the‐art Haber–Bosch process, which spans 9.5–10.5 kWh kg^−1^.^[^ ^59^ ^]^ If we take a lower limit of energy consumption in the Haber‐Bosch process as reference, then voltage that is larger than 2.0 V is not likely to be competitive. Considering the commercial price of 1 kg of NH_3_, which is around 0.46 Euro,^[^ ^60^ ^]^ if electrolysis would be conducted at a realistic voltage of 2 V, the acceptable price for kWh of electricity should be below 0.050 Euro. This actually makes electrolytic synthesis of NH_3_ more challenging than the synthesis of C_3_H_7_OH, while less challenging compared to CH_4_, C_2_H_4_, and CH_3_OH. A major issue with NH_3_ is that activation of very inert N_2_ molecule is extremely challenging,^[^ ^61^ ^]^ even more demanding than activation of CO_2_ molecule,^[^ ^62^, ^63^ ^]^ therefore, FE is very low.^[^ ^64^ ^]^ Also, applied current densities for NH_3_ electrosynthesis are low, and/or operational voltages are very high.^[^ ^65^ ^]^ Overall this suggests that, despite ammonia exhibits some interesting properties (e.g., can be liquified much easier than hydrogen), it is not likely to be a recommended strategy for renewable electricity storage (**Figure** **3**).

### 2.4 What would be More Realistic Scenarios?

We estimated for all the analyzed energy storage media, the upper limit for the acceptable price of electricity. It is a value for which price of electricity used for electrolysis at realistic operational voltage is approximately equal to the market price of the energy medium. For H_2_ generation from water, and for all the products of CO_2_ electrolysis that require the transfer of more than two electrons and two protons, except C_3_H_7_OH, as well as for NH_3_ electrosynthesis from elemental nitrogen, the upper limit for acceptable electricity price is in the range of existing prices of renewable electricity generated from PV, that is currently around 0.04 Euro per kWh. If we take, for example, into consideration renewable H_2_ production where electricity contributes to ≈50% to the overall costs,^[^ ^66^, ^67^ ^]^ including CAPEX (capital expenditures), other OPEX (operational expenditures), grid fees, and taxes, we would end up with a price of the product that is still far away from competitive. It is even more delicate in the case of CO_2_ electrolysis^[^ ^68^ ^]^ (i.e., excluding HCOOH and CO) or green NH_3_ electrosynthesis,^[^ ^69^ ^]^ where electricity is also around 50% of the overall costs, taking into consideration that selectivity in the case of some products is significantly below 100%, that utilized current densities were relatively low as well as the fact that the operating voltages in some cases could be higher.^[^ ^70^ ^]^ All that would add to the cost of electricity for 1 kg of product, cost for product separation, required surface area of electrodes, etc. From here on, it is important to understand that if the cost of electricity is around 50% of overall production costs, and that is approximately equal to the market price of the energy storage medium, then we need to consider what can be realistically done to further reduce the production costs by ≈50%. If one analyzes the structure of CAPEX (stack, balance of plant, and other utilities, etc.),^[^ ^71^ ^]^ in our opinion, it does not offer the possibility for drastic improvements in the future. Certainly, it is important that the entire system where electrolysis is performed is stable^[^ ^72^ ^]^ and uses reliable equipment^[^ ^73^ ^]^ to respond to renewable energy source intermittency and to prevent any significant decay in energy conversion efficiency.^[^ ^74^ ^]^ If the electrolyzer and surrounding infrastructure can function reliably for thousands of hours, that will additionally reduce CAPEX and shift focus on OPEX, especially toward reducing the cost of electricity consumed for electrolysis. On the one hand, it would be certainly necessary, for the defined range of current density (e.g., 300–500 mA cm^−2^) to enhance selectivity of the reactions, but on the other hand, it is even more important to significantly reduce the overpotential (e.g., especially in the case of CH_4_).^[^ ^75^ ^]^ The second option would be to enhance the efficiency of PVs so that renewable electricity prices drop significantly. In quantitative terms, if we need to reduce the production costs by ≈50%, the price of renewable electricity has to go from around 0.04 Euro per kWh to below 0.02 Euro per kWh. A drop in price of renewable electricity to 0.02 Euro per kWh would be equivalent to reducing the reactor voltage by ≈50%, which would mean that we perform electrolysis at the theoretical or very close to the theoretical voltage (with the exception of CH_4_), which is practically not feasible. As stated previously, a uniquely different situation applies to HCOOH and CO (or eventually syngas). At the same time, despite the conversion of CO_2_ to HCOOH and CO being economically feasible, it is worth to mention that global production of these two products as well as CH_4_ and C_3_H_7_OH is relatively small (i.e., each of them on the scale of several billions of Euro annually) while gigantic markets are for CH_3_OH, C_2_H_5_OH and C_2_H_4_, reaching over 50 billion, 100 billion and 200 billion Euro, respectively.^[^ ^76^ ^]^ It is interesting, as previously stated, that the acceptable price of 1 kWh of electricity for the production of C_3_H_7_OH would be two times higher than for the other analyzed products of CO_2_ electrolysis, at high current densities, as well as significantly enhanced selectivity.^[^ ^52^ ^]^ The question is whether it is a coincidence or indeed higher alcohols or hydrocarbons would be economically feasible to produce if we obtain knowledge on how to control the selectivity and electrocatalytic activity of electrochemical CO_2_ reduction.

It is important to note that when we discuss energy consumption during electrolytic processes, it is always, by default, identified with the operating voltage. However, the energy required to perform water electrolysis, CO_2_ electrolysis, or electrosynthesis of NH_3_ depends on voltage and charge used for electrolysis, where charge used for electrolysis depends on the ratio between molar mass and the number of exchanged electrons to generate one mole of the product. The ratio of molar mass to number of exchanged electrons (*M*/*n*_e‐_), together with parameters determining efficiency of processes for the analyzed energy storage media, is given in Table 1. Important to note from Table 1, when comparing the contribution of voltage to the value of energy stored in 1 kg of a given medium with the contribution of the charge used for electrolysis, is that the contribution of the latter is, in quantitative terms, significantly larger. Taking into consideration that for all the analyzed reactions the theoretical voltage is similar to the voltage for water splitting (i.e., 1.23 V) or, to be precise, in the range of 1.23 ± 0.2 V, the essential contributor to the theoretical amount of energy that can be stored in 1 kg of medium is *M*/*n*_e‐_. Whereas the theoretical voltage change for the analyzed products spans in the range of ±16%–17%, the *M*/*n*_e‐_ spans from 1 (H_2_) or 2 (CH_4_) up to 23 (HCOOH).

**Figure 4:**
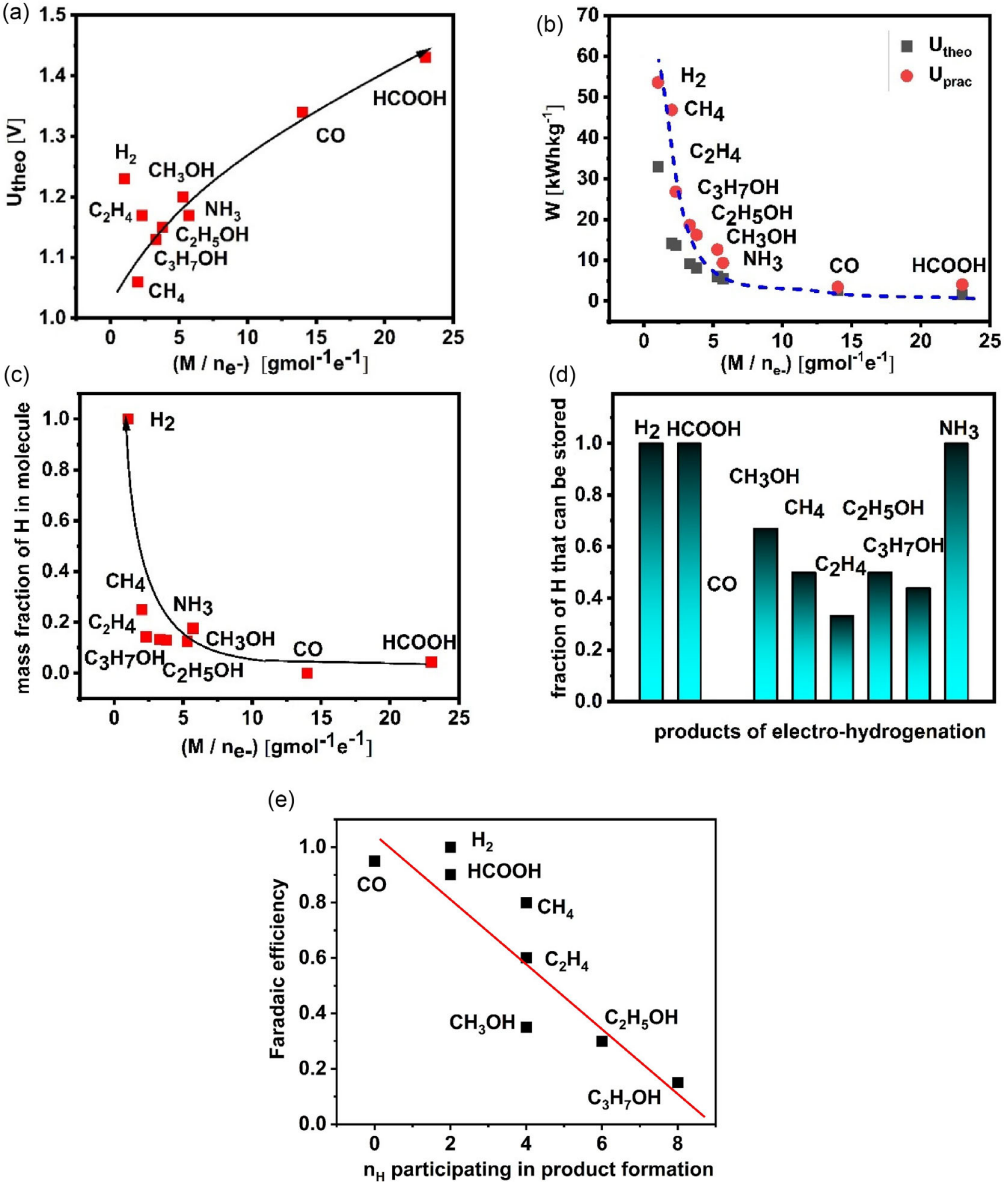
Parameters determining the amount of energy stored per unit mass as well as parameters impacting several aspects of conversion efficiency: a) relation between theoretical voltage (*U*_theo_) and *M*/*n*_e‐_ ratio, b) impact of *M*/*n*_e‐_ on theoretical energy stored per unit mass, c) impact of the *M*/*n*_e‐_ ratio on mass percent of hydrogen in molar mass of energy storage medium, d) maximal percent of hydrogen or (H^+^ + e^−^) generated at anode by water oxidation that can be converted to storage medium, and e) impact of the number of hydrogen atoms (H^+^ + e^−^) that become an integral part of molecule of the product on faradaic efficiency.

In **Figure** **4**a, we can observe that the theoretical voltage and the *M*/*n*_e‐_ are positively correlated, and consequently, the minimum amount of energy that needs to be invested for storage in 1 kg of generated medium depends on the ratio between theoretical *U*_theo_ and *M*/*n*_e‐_, where *M*/*n*_e‐_ has a profound impact on *W*_theo_. (Figure 4b). If one carefully examines Figure 4a ,4b, it can be concluded that the increasing trend in energy density, which is inversely proportional to *M*/*n*_e‐_ (Figure 4b), is, at the same time, followed by the drop in the voltage (Figure 4a). In other words, the energy density (Table 2) for the studied energy storage media is increasing, despite voltage drops, due to a very emphasized increase in charge (or drop in *M*/*n*_e‐_). Therefore, it is very relevant to understand how the *M*/*n*_e‐_ ratio impacts energy density. This is straightforward only if we take into consideration energy density based on the unit of mass (i.e., gravimetric energy density). If we utilize volumetric energy density (Table 2), we cannot see relation analogue to relation shown in Figure 2b. This is due to the fact that volumetric energy densities of gaseous products (H_2_, CO, CH_4_, C_2_H_4_, and NH_3_) have very low density, resulting in very low volumetric energy density.

When we analyze contribution of hydrogen to the total molar mass of molecule used as storage medium versus *M*/*n*_e‐_, we can see that they are negatively correlated (Figure 4c). Electrocatalytic hydrogenation (i.e., transfer of proton/electron couple) will proceed to a larger extent, for storage media that have low *M*/*n*_e‐_ (Figure 4e). It is interesting to note from the stoichiometry of the reactions that for some of the molecules which were used as storage media, a significant portion of the proton/electron couples are not used for hydrogenation, but rather for water formation. The exception to this is evidently H_2_, HCOOH, and NH_3_. Among the carbon‐based media, methanol exhibits the highest utility of the proton‐electron (H^+^ + e^−^) couples generated at anode by water oxidation, close to 67%. For methane and ethanol, utility of the (H^+^ + e^−^) couples is 50% while for propanol and ethylene, it is below 50%, being around 44% and around 33%, respectively. Finally, during CO synthesis, all (H^+^ + e^−^) couples are transferred to produce water (Figure 4d).

From all this, the following can be anticipated: 1) Despite the relatively low amount of electricity stored per unit of mass of HCOOH and CO in comparison to the other analyzed carbon‐containing media or ammonia, the‐electrocatalytic generation of HCOOH and CO is evidently far less challenging than for the other studied storage media, and possible to be achieved at the prevailing electricity prices. 2) The energy efficiency (Table 1) of H_2_ and CO is higher in comparison to other studied storage media. H_2_ is generated at much higher current densities and similar voltages like CO. Further work is necessary, despite high selectivity, to reduce voltage and enhance current density for HCOOH. For other storage media, it would be a progressive step forward if stable conditions of operation could be achieved, assuming an operational voltage of 2 V, current density of 300 mA cm^−2^, with selectivity above 90%. 3) Despite water electrolysis being the most advanced and the most important technology, it is still too expensive to generate H_2_ at market‐competitive prices, especially if H_2_ is used as a building block for further synthesis of more complex chemical compounds. Advances and breakthroughs in electrocatalysis and electrochemical engineering can certainly contribute to further efficiency improvement and price reduction, however, parallel, and not less important, effort should be in the direction of the physics of semiconductors to improve the efficacy and durability of PVs. Desired outcome of PV improvement will be a reduction in price of renewable electricity to 50% (i.e., to reach price below 2 Eurocent for 1 kWh). 4) The *M*/*n*_e‐_ ratio has a much more profound impact on the theoretical energy density of a given energy storage medium than voltage (Figure 4a,b). Furthermore, the percent of hydrogen with respect to the molar mass of the energy storage medium increases with a decrease in the *M*/*n*_e_ (Figure 4c). Despite the percent of hydrogen with respect to the molar mass of a given energy storage medium being higher for media with a low *M*/*n*_e‐_ ratio, they exhibit significantly more complicated selectivity control. 5) Selectivity was inversely related to the number of hydrogen atoms that really become an integral part of newly generated molecule of the storage medium (Figure 4e). As the number of hydrogen atoms (H^+^ + e^−^) involved in the hydrogenation process increases—illustrated by the comparison between CH_3_OH, C_2_H_5_OH, and C_3_H_7_OH in Figure 4d—the proportion of hydrogen atoms consumed in water formation at the cathode rises steadily, relative to the proportion that is actually incorporated into the target storage molecule.

For example, at the anode, out of 9 water molecules required to generate 18 hydrogen atoms (H^+^ + e^−^) that will be consumed for generation of C_3_H_7_OH at cathode, 8 hydrogen atoms participate in product formation, while 10 end up forming water at the cathode. In other words, out of 9 water molecules splatted at the anode, 4 ends up in energy storage medium while 5 are regenerated at the cathode. First, a nontrivial fact can be observed here that electrochemical CO_2_ reduction consumes substantial amount of water. Second and more important is that the energy is consumed at the anode, to split 9 water molecules, while hydrogen atoms (H^+^ + e^−^) originating from only 4 water molecules will build the product. Finally, in order to generate one molecule of C_3_H_7_OH, it is necessary to split 9 water molecules, and then protons need to diffuse and migrate and participate in transformation of CO_2_ to C_3_H_7_OH. This means that minimizing of overpotential for CO_2_ reduction should be prioritized only if kinetics of CO_2_ is more sluggish than, as described above, process of splitting of 9 water molecules, combined with transport of protons toward the cathode. Our intuition suggests that for the generation of molecule like C_3_H_7_OH, it is very probable that entire kinetics of the system is controlled significantly by the anode reaction and that reduction of overpotential is probably related more to OER than to CO_2_ electroreduction. At the same time, tuning of selectivity at the cathode is one of the central challenges that will practically shape further developments in the field.

From stated points above, it is evident that H_2_, HCOOH, and CO (or syngas) synthesis standout as the most promising energy storage media that deserve special focus. The other storage media where more than two electrons are exchanged exhibit low energy efficiency and poor selectivity despite having high theoretical energy densities and containing a relatively high percentage of hydrogen. The low energy density and poor content of hydrogen in HCOOH and syngas can be compensated with high product turnover by operating at higher current densities. This seems to be a much more realistic expectation than in the case of media requiring the exchange of more than two electrons and protons, characterized with higher overpotentials at relatively low current densities, poor selectivity control, and unrealistically low electricity pricing requirements. At the end, it is worth to have in mind that economic encouragement of green transition via carbon tax and similar measures could impact the market of energy storage media, however, there is also the possibility that carbon tax will never be applied. From that perspective, our efforts should not diminish, because we live in a world of limited resources that require preservation of the environment where very important segment is clean and sustainable utilization of energy and raw materials.^[^ ^77^ ^]^

## 3 Conclusion

The analysis of electricity‐to‐chemical conversion for selective electrochemical reduction of CO_2_ to specific molecules, water to hydrogen and nitrogen to ammonia, was conducted, taking into consideration the basic production parameters, including the actual price of renewable electricity and the price of industrial electricity, voltage of the reactor and the mass to charge ratio of the molecules, among others, to determine the chemical energy storage media that are more realistic and promising to be implemented in the immediate future for storage of renewable electricity. Products that require more than two electrons and protons, despite their ability to store a substantial amount of renewable electricity per unit mass (i.e., theoretical energy density), require unrealistically low cost of electricity, have too low energy efficiency and exhibit serious challenges in control of selectivity, especially if electrolysis is to be conducted on extended timescale. The results of our simple techno‐economic analysis revealed that the production of H_2_, HCOOH, and CO (or even better syngas) is promising, and thus, targeting these molecules in the short‐term provides a rational and more realistic solution. Worth to mention is that for H_2_ and HCOOH, practically 100% of all the protons that are delivered to the cathode are stored in the generated molecule, and no protons delivered from the anode are consumed for water formation. Despite some of the analyzed carbon‐based storage media having high energy density comparable or better than gasoline (e.g., propanol), there is no evident promise of economic viability. Therefore, it would be more realistic to produce H_2_, HCOOH, CO, and syngas, and then use them as building blocks for synthesis of more complex compounds through classical thermal catalysis procedures. The existing infrastructure of the current chemical industry has been built in a period of more than 150 years with vast accumulated experience, therefore, phasing out of such well‐established technologies should be conducted carefully, especially, in the light of implementing new technologies that are still in their infancy with numerous open questions.

## Conflict of Interest

The authors declare no conflict of interest.

## Author Contributions

**Jacob Johny**: co‐writing draft, literature discussion, draft corrections. **Sayed M. El‐refaei**: literature discussion, draft corrections. **Justus Masa**: literature discussion, draft corrections, language editing. **Aleksandar R. Zeradjanin**: design of study, literature overview and discussion, writing draft, draft corrections.

## Data Availability

The data that support the findings of this study are available from the corresponding author upon reasonable request.
